# Clinicopathologic characteristics and diagnostic value of PD-L1 in immune checkpoint inhibitor-related liver injury: a single-center retrospective study

**DOI:** 10.3389/fonc.2026.1928835

**Published:** 2026-10-07

**Authors:** Huaying Lai, Xinyan Zhao, Dongmei Zhou, Luping Lin, Jianwei Zhang, Yu Chen, Sha Huang

**Affiliations:** 1 Department of Medical Oncology, Clinical Oncology School of Fujian Medical University, Fujian Cancer Hospital, Fuzhou, China; 2 Liver Research Center, Beijing Friendship Hospital, Capital Medical University & National Clinical Research Center for Digestive Diseases, Beijing, China; 3 Department of Pathology, Clinical Oncology School of Fujian Medical University, Fujian Cancer Hospital, Fuzhou, China

**Keywords:** biomarker, characteristics, hepatotoxicity, immune checkpoint inhibitor, immunotherapy, liver injury, prognosis

## Abstract

**Background and aims:**

Immune-mediated liver injury caused by checkpoint inhibitors (ILICI) differs from chemotherapy- or targeted drug-related hepatotoxicity in pathogenesis and management. Biomarkers to distinguish ILICI from other anticancer agent-induced liver injuries are lacking. We compared the clinicopathological features, treatments, and outcomes of ILICI versus other anticancer agent-related liver injuries to identify distinguishing biomarkers.

**Methods:**

We retrospectively included patients with liver injury during anticancer therapy at Fujian Cancer Hospital between June 2022 and November 2024. Cases were classified as ILICI or non-ILICI using the Common Terminology Criteria for Adverse Events v5.0 and the Roussel Uclaf Causality Assessment Method.

**Results:**

Sixty-seven patients were enrolled, including 30 ILICI and 37 non-ILICI. ILICI was associated with more high-grade liver injury than non-ILICI (*P* = 0.001), with similar latency, recovery time, and prognosis. Corticosteroids were administered in 70% of ILICI vs. 5.4% of non-ILICI patients (*P* < 0.001). Among 29 liver biopsies (19 ILICI, 10 non-ILICI), ILICI showed a higher focal necrosis score (*P* = 0.031) and programmed death-ligand 1 (PD-L1) expression in liver sinusoidal endothelial cells (LSECs), lymphocytes, and Kupffer cells (KCs) (*P* = 0.007). Receiver operating characteristic analysis showed that a PD-L1 positivity cutoff of >30% in LSECs, lymphocytes, and KCs discriminated ILICI with an AUC of 0.811 (95%CI: 0.641–0.980). Sensitivity analyses and Firth penalized logistic regression confirmed that this finding remained robust after adjustment for confounders including pre-biopsy corticosteroid use and inactive HBV carrier status.

**Conclusions:**

ILICI was associated with more severe hepatic inflammatory necrosis and a greater need for immunosuppressive therapy than non-ILICI. The PD-L1 positivity rate >30% in LSECs, lymphocytes, and KCs may serve as a potential diagnostic biomarker for ILICI.

## 1 Introduction

Recently, immune checkpoint inhibitors (ICIs) have shown good efficacy in various malignant tumors, such as malignant melanoma, gastric cancer, and liver cancer, leading to their increasing clinical application (1–4). While ICIs enhance the host’s antitumor activity by blocking immune checkpoints, they may also cause immune-related adverse events (irAEs) affecting several organs such as the skin, endocrine system, respiratory system, gastrointestinal tract, and liver. Of these, immune-mediated liver injury caused by checkpoint inhibitors (ILICI) is one of the most common adverse reactions. Its occurrence often leads to the temporary or permanent discontinuation of antitumor drugs (5), delaying further anticancer treatment and potentially causing disease progression and shortened survival. The incidence of liver injury resulting from ICI monotherapy was 2% to 10% (6, 7), with a 1% to 2% incidence of high-grade liver injury that was classified as grade 3–5 hepatitis in accordance with Version 5.0 of the Common Terminology Criteria for Adverse Events (CTCAE v5.0) (8). Although the mortality rate of ILICI was lower than that of immune-related colitis, pneumonia, and myocarditis, according to a systematic review and meta-analysis, ILICI still accounted for 8% to 11% of ICI-related deaths in the same cohort (9).

ILICI differs fundamentally in pathogenesis from liver injury caused by other anticancer agents: it is immune-mediated (10), whereas the latter typically results from direct cytotoxicity, reactive metabolites, oxidative stress, or mitochondrial dysfunction (11–13). Given these inherent pathogenic differences, the management strategies do so too (13). As novel anticancer drugs emerge, combination regimens have become standard for many advanced malignancies to maximize efficacy. Therefore, when liver injury occurs, identifying the causative agent is challenging, and pinpointing the hepatotoxic drug is crucial for individualized treatment. Comparative data on liver injury associated with immunotherapy versus non-immunotherapy remain limited. Previous studies have chiefly reported crude differences in hepatotoxicity incidence or severity between chemotherapy and immunotherapy (14, 15), without comprehensive analyses of clinicopathological features, management, or outcomes, and reliable diagnostic biomarkers are still lacking.

Therefore, this study aimed to compare the clinical and pathological characteristics, treatment, and prognosis of liver injury caused by ICIs and other anticancer drugs, and to find potential diagnostic biomarkers that can distinguish between the drugs causing liver injury. This is meaningful for making an accurate diagnosis and guiding subsequent treatment.

## 2 Patients and methods

### 2.1 Study design and patients

We retrospectively included patients who developed liver injury during anticancer treatment at Fujian Cancer Hospital from June 2022 to November 2024. Liver injury was defined as grade 1 or higher liver function abnormalities, including elevated serum levels of alanine transaminase (ALT), aspartate transaminase (AST), alkaline phosphatase (ALP), and total bilirubin (TBIL), according to CTCAE v5.0 (16). When a patient was receiving multiple medications, the Roussel Uclaf Causality Assessment Method (RUCAM), which classifies the likelihood of drug-induced liver injury as highly probable (>8 points), probable (6–8 points), possible (3–5 points), or unlikely (1–2 points), was used to clarify the causal relationship between the suspected drug and the liver injury. Those with a RUCAM score of 6 or higher, or with a RUCAM score of 3–5 and a diagnosis agreed upon by two liver specialists, were included in the study. RUCAM scoring was jointly performed by two clinicians with 5 years of experience in hepatology based on medical records. For patients receiving multiple anti-tumor agents, independent RUCAM scores were calculated for each drug, and the highest single-drug score was taken as the patient’s RUCAM score. A drug with a RUCAM score ≥6 was considered a culprit hepatotoxic drug; if multiple drugs scored ≥6, the liver injury was attributed to a combined effect of multiple agents. For patients with a RUCAM score of 3–5, both the diagnosis of drug-induced liver injury and the culprit drug had been confirmed during prior clinical management, and were only retrospectively collected in this study.

Exclusion criteria included patients with liver injury attributable to any of the following conditions: acute viral hepatitis A, B, C, or E; uncontrolled chronic hepatitis B or C; non-hepatotropic viral infections such as cytomegalovirus, herpes simplex virus, or Epstein–Barr virus; autoimmune hepatitis; primary biliary cholangitis (PBC); primary sclerosing cholangitis; alcoholic liver disease; non-alcoholic steatohepatitis (NASH); ischemic or hypoxic liver injury; Wilson’s disease; biliary obstruction; progressive hepatic malignancy; or hepatotoxicity induced by non-antineoplastic medications. We also excluded patients taking multiple hepatotoxic drugs with unclear causality, as well as those with incomplete baseline data or loss to follow-up. All hepatitis B surface antigen (HBsAg)-positive patients received antiviral treatment with nucleoside analogues during anticancer therapy. All HBsAg-positive patients with elevated ALT and/or AST levels underwent hepatitis B virus (HBV) DNA quantitative assessment to rule out HBV reactivation.

Follow-up of all enrolled patients was conducted through a systematic review of electronic medical records. The observation period began at the initial documentation of liver injury. Primary outcomes were defined as follows: (1) liver function tests (LFTs) returned to normal or baseline levels; (2) chronicity, characterized by persistent elevation of LFTs for ≥ 6 months; (3) liver failure; (4) liver transplantation; and (5) all-cause mortality. The secondary outcome was recovery of LFTs to CTCAE grade 1.

The study was conducted in accordance with the Declaration of Helsinki and approved by the Ethics Committee of Fujian Cancer Hospital (No: K2025-325-01).

### 2.2 Clinical data

This study collected clinical data on patients, including gender, age, clinical symptoms, underlying liver disease, extrahepatic underlying disease, alcohol consumption history, smoking history, allergy history, tumor type, tumor stage, Eastern Cooperative Oncology Group Performance Status (ECOG-PS) score, antitumor therapeutic schedule, concomitant medication, drugs causing liver injury, time of liver injury onset, treatment, and clinical outcome.

Upon the onset of liver injury, comprehensive laboratory and imaging evaluations were performed to exclude alternative etiologies and to grade injury severity. Laboratory tests included serum ALT, AST, ALP, γ-glutamyl transferase (GGT), TBIL, direct bilirubin (DBIL), prothrombin time, immunoglobulin (Ig) M and IgG, autoantibody profiling (including anti-mitochondrial antibody [AMA], AMA-M2, anti-gp210, anti-sp100, antinuclear antibody, anti-smooth muscle antibody, anti-liver–kidney microsomal antibody type 1, anti-liver cytosol antigen type 1, and anti-soluble liver antigen/liver-pancreas antigen), and serologic markers for hepatitis A, B, C, and E viruses, herpes simplex virus, cytomegalovirus, and Epstein–Barr virus.

### 2.3 Histological evaluation

Liver tissue sections were stained with hematoxylin and eosin (H&E), reticulin, Masson’s trichrome, periodic acid–Schiff (PAS), diastase-PAS (D-PAS), Prussian blue, rhodanine, aldehyde fuchsin, HBsAg, hepatitis B core antigen, cytokeratin (CK) 7, CK19, cluster of differentiation (CD) 3, CD4, CD8, CD20, multiple myeloma oncogene 1, programmed cell death-1 (PD-1), and programmed death-ligand 1 (PD-L1). All liver biopsies were re-evaluated independently by two hepatic pathologists blinded to the final diagnosis; discrepancies were resolved by joint review to reach consensus.

All liver tissue sections were evaluated for the following histological features and graded according to the Ishak scoring system (17): focal necrosis (0–4 points), confluent necrosis (0–6 points), bridging necrosis, portal inflammation (0–4 points), interface inflammation (0–4 points), ductular reaction, cholestasis, bile duct damage, fibrosis (0–6 points), perisinusoidal fibrosis, steatosis, and endotheliitis. Liver injury patterns were classified into acute hepatitis, acute cholestasis, chronic hepatitis, chronic cholestasis, cholestatic hepatitis, and other types (18).

PD-L1 immunohistochemistry was performed using the PD-L1 (28-8) antibody clone on the Dako Autostainer Link 48 platform (Agilent Technologies, Santa Clara, CA, USA). PD-L1 staining intensity was scored on a semiquantitative scale: 0 (negative, no membranous staining), 1+ (weak, partial membranous staining), 2+ (moderate, clear membranous staining), and 3+ (strong, dark and uniform membranous staining). Only membranous staining was evaluated, and cells with intensity ≥1+ and definitive membranous staining were defined as positive. Five non-overlapping high-power fields (×400) were selected per slide. Selected fields were enriched for liver sinusoidal endothelial cells (LSECs), lymphocytes and Kupffer cells (KCs), while necrotic, hemorrhagic, and severely fibrotic areas were avoided. PD-L1 positivity was quantified as the percentage of area within selected high-power fields occupied by membranous PD-L1–positive LSECs, lymphocytes, and KCs. Staining intensity grade and positivity grade were independently and blindly scored by two liver pathologists with 5 years of experience using manual estimation. The final quantitative positivity rate was determined by joint review and consensus between the two pathologists.

### 2.4 Statistical analysis

Quantitative variables were expressed as mean ± standard deviation or median (interquartile range [IQR]) and compared using the t-test or Mann-Whitney U test. Qualitative data were expressed as numbers (percentages) and compared using the chi-square test or Fisher’s exact test. Receiver operating characteristic (ROC) curve analysis was used to determine the optimal cutoff value, and 0.632 bootstrap internal validation with 1000 resamples was performed to correct optimism bias, with sensitivity analyses performed to assess the robustness of the results. Firth penalized logistic regression was used to adjust for confounders. Weighted kappa was used to evaluate inter-observer agreement between two observers for PD-L1 scoring. Spearman correlation was used to assess the association between the CD8^+^T/CD3^+^T ratio and PD-L1 positivity. Firth penalized logistic regression, weighted kappa and 0.632 bootstrap internal validation were performed using R software (version 4.5.3). All other statistical analyses were performed using SPSS version 25 (IBM Corp., Chicago, IL, USA). *P* < 0.05 was considered statistically significant.

## 3 Results

### 3.1 Patient characteristics at baseline

Sixty-seven patients were enrolled: 30 with ILICI and 37 with non-ILICI (**Figure 1**). Baseline characteristics of all participants are summarized in **Table 1**. The median age was 58 years (IQR, 51–66), and 64.2% of the patients were male. Thirteen patients had complicated HBV infection, and hepatitis B reactivation was excluded in all cases upon liver injury occurrence. The most common type of tumor was gastrointestinal malignancies, followed by malignant melanoma. The majority of patients presented with advanced disease (Tumor-Node-Metastasis stage III–IV) and maintained a good performance status (ECOG-PS 0–1). No significant inter-group differences were observed for gender, age, smoking, alcohol use, comorbidities (including hypertension and diabetes), tumor type, tumor stage, or ECOG-PS. Baseline median LFTs were within normal limits in both groups.

**Figure 1:**
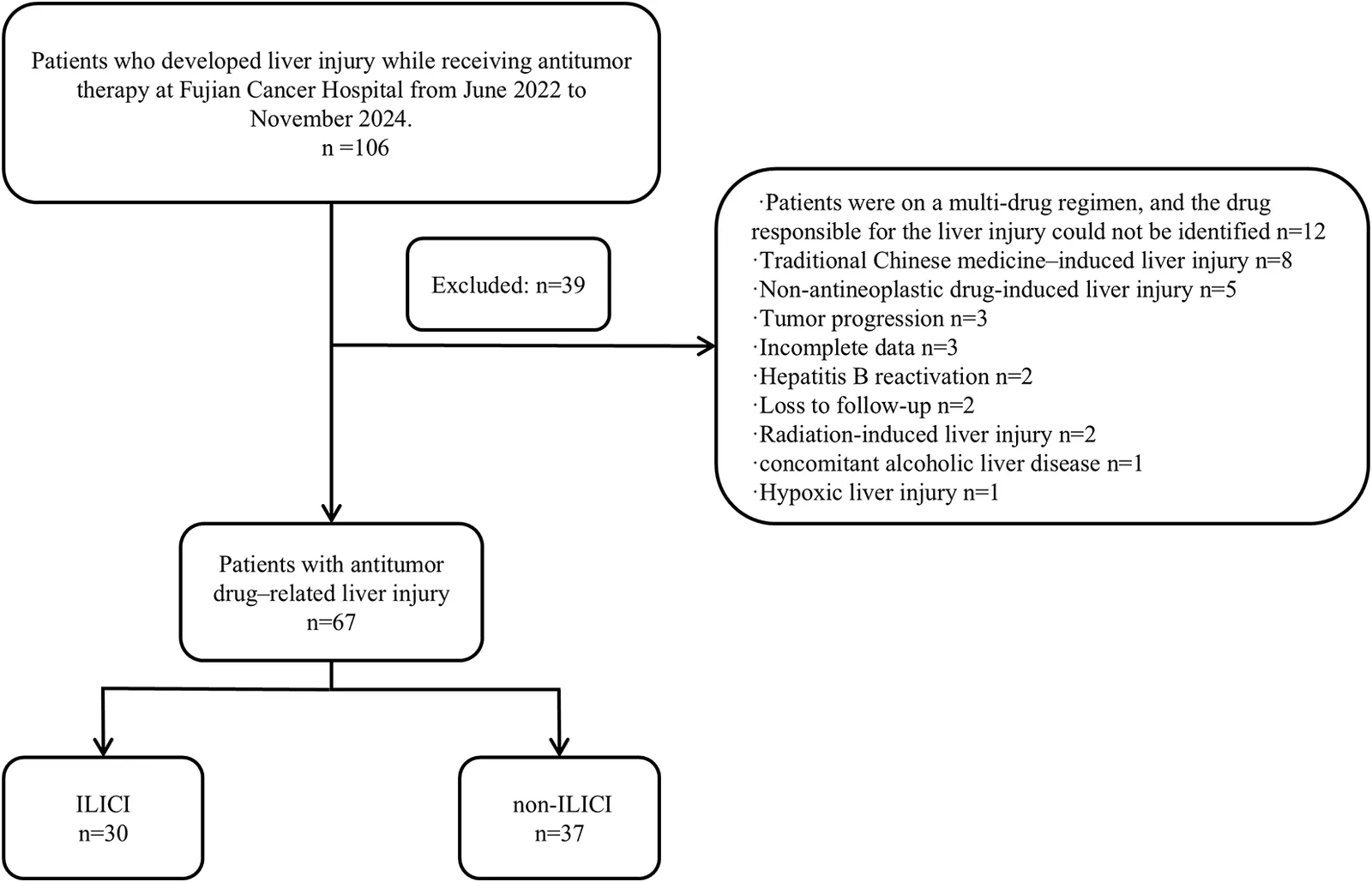
Flowchart of included patients. ILICI, Immune-mediated liver injury caused by checkpoint inhibitors.

**Table 1:** Patient characteristics at baseline.

Clinical characteristics	All patient(n=67)	ILICI(n=30)	Non-ILICI(n=37)	*P*-value
Age (years), median (IQR)	58(51, 66)	59.5(51.8, 68.5)	58(48.5, 64.5)	0.185
Gender (male)	43(64.2%)	23(76.7%)	20(54.1%)	0.055
Drinking history	17(25.4%)	5(16.7%)	12(32.4%)	0.140
Smoking history	19(28.4%)	9(30%)	10(27.0%)	0.788
Hypertension	16(23.9%)	7(23.3%)	9(24.3%)	0.925
Diabetes	8(11.9%)	2(6.7%)	6(16.2%)	0.412
Previous hepatic disease				
HBV	13(19.4%)	11(36.7%)	2(5.6%)	0.002
NAFL	6(9.0%)	4(13.3%)	2(5.4%)	0.484
Compensated cirrhosis	4(6.0%)	3(10.0%)	1(2.7%)	0.462
Liver tumor*	29(43.3%)	10(33.3%)	19(51.4%)	0.139
Cancer types				0.100
Gastrointestinal cancer	30(44.8%)	9(30.0%)	21(56.8%)	
Melanoma	11(16.4%)	6(20.0%)	5(13.5%)	
Primary liver cancer	6(9.0%)	5(16.7%)	1(2.7%)	
Lung cancer	6(9.0%)	4(13.3%)	2(5.4%)	
Other cancers	14(20.9%)	6(20.0%)	8(21.6%)	
Tumor stage (TNM stage)				0.197
II	2(3.0%)	2(6.7%)	0(0%)	
III-IV	65(97.0%)	28(93.3%)	37(100%)	
ECOG-PS				0.448
0-1	66(98.5%)	29(96.7%)	37(100%)	
2	1(1.5%)	1(3.3%)	0(0%)	
RUCAM score				0.646
3-5	19(28.4%)	10(33.3%)	9(24.3%)	
6-8	42(62.7%)	17(56.7%)	25(67.6%)	
>8	6(9.0%)	3(10.0%)	3(8.1%)	
Culprit anticancer agent				—
ICI monotherapy	30(44.8%)	30(100%)	0(0%)	
Chemotherapy monotherapy	19(28.4%)	0(0%)	19(51.4%)	
Targeted therapy monotherapy	11(16.4%)	0(0%)	11(29.7%)	
Antibody–drug conjugate monotherapy	3(4.5%)	0(0%)	3(8.1%)	
Targeted therapy and chemotherapy	4(6.0%)	0(0%)	4(10.8%)	
Liver function tests				
ALT(U/L)	21(14, 36)	24.5(14.8, 41.5)	20.0(11.5, 32.0)	0.328
AST(U/L)	27(21, 39)	26.0(19.0, 39.8)	27.0(22.0, 37.0)	0.686
ALP(U/L)	98(74, 136)	107.5(71.5, 162.8)	96.0(79.5, 126.5)	0.614
GGT(U/L)	37(23, 98)	37.0(23.5, 166.0)	34.0(22.5, 73.5)	0.609
TBIL(μmol/L)	11.4(9.6, 14.4)	10.6(9.4, 14.5)	12.0(9.7, 13.1)	0.541
DBIL(μmol/L)	2.3(1.9, 3.1)	2.5(1.9, 3.5)	2.3(1.8, 3.0)	0.557

*: Liver tumors include primary liver cancer and liver metastases.

ILICI, immune-mediated liver injury caused by checkpoint inhibitors; IQR, interquartile range; ICI, immune checkpoint inhibitor; HBV, hepatitis B virus; NAFL, Non-alcoholic fatty liver; TNM stage, Tumor-Node-Metastasis stage; ECOG-PS, Eastern Cooperative Oncology Group Performance Status; RUCAM, Roussel Uclaf Causality Assessment Method; ALT, alanine-aminotransferase; AST, aspartate-aminotransferase; ALP, alkaline phosphatase; GGT, gamma-glutamyl transpeptidase; TBIL, total bilirubin; DBIL, direct bilirubin.

Percentages may not total 100 because of rounding.

### 3.2 Patient characteristics at the time of liver injury

Latency, administered cycles, and concomitant irAEs did not differ between the two groups. Peak levels of ALT, AST, TBIL, ALP, and GGT were all higher in ILICI patients than in non-ILICI patients, with the first three differences reaching statistical significance (*P* < 0.05), and 83.3% of ILICI cases were classified as high-grade (grade 3–5) liver injury compared with 56.8% of non-ILICI cases that were low-grade (grade 1–2) liver injury. Times to peak values for ALP, GGT, and TBIL were shorter in the ILICI group, although only TBIL showed a statistically significant difference (*P* =0.008), whereas ALT and AST peak times were similar between groups (**Table 2**).

**Table 2:** Patient characteristics at the time of liver injury.

Clinical characteristics	ILICI (n=30)	Non-ILICI (n=37)	*P-*value
Latency*(days)	30.5(14.0, 59.5)	36.0(21.0, 69.0)	0.427
Number of cycles administered	2.0(1.0, 2.3)	1.8(1.0, 2.7)	0.779
Peak liver enzyme level			
ALT(U/L)	353.0(172.5, 550.3)	156.0(105.5, 283.5)	0.005
AST(U/L)	362.0(213.5, 500.5)	117.0(78.0, 184.0)	<0.001
ALP(U/L)	224.5(153.1, 333.5)	153.0(111.5, 268.0)	0.121
GGT(U/L)	257.0(167.3, 438.0)	206.0(66.0, 442.5)	0.217
TBIL(μmol/L)	30.7(19.7, 58.7)	14.5(11.0, 22.2)	<0.001
Severity of hepatotoxicity (CTCAE grade)			
Grade 1-2	5(16.7%)	21(56.8%)	0.001
Grade 3-5	25(83.3%)	16(43.2%)	
Time to peak ^#^(days)			
ALT	7.0(0, 18.8)	7.0(0, 29.0)	0.856
AST	7.5(0, 18.8)	7.0(0, 29.0)	0.776
ALP	8.5(2.8, 15.8)	22.0(2.5, 35.5)	0.059
GGT	14.0(2.0, 30.3)	17.0(5.5, 41.0)	0.365
TBIL	8.5(0.8, 21.0)	26.0(6.5, 69.5)	0.008
Concomitant irAEs in other organs	6(20.0%)	15(40.5%)	0.072

*: The latency was the interval between first drug exposure and onset of liver injury.

#: Time to peak was defined as the interval from the onset of liver injury to the peak liver enzyme level.

ILICI, immune-mediated liver injury caused by checkpoint inhibitors; CTCAE, Common Terminology Criteria for Adverse Events; ALT, alanine-aminotransferase; AST, aspartate-aminotransferase; ALP, alkaline phosphatase; GGT, gamma-glutamyl transpeptidase; TBIL, total bilirubin; irAEs, immune-related adverse events.

### 3.3 Histological and immunohistochemical characterization

Liver biopsy was obtained in patients with unexplained liver injury, persistently worsening liver function during conventional treatment, or recurrence during corticosteroid tapering. Liver biopsies were performed on 29 patients, including 19 ILICI and 10 non-ILICI cases. Of these, five patients had concomitant HBV infection, but all were in the inactive phase of HBsAg carriage. Eleven patients had already received corticosteroids before biopsy: four for preceding irAEs (nephritis, hypophysitis, or rash) and seven for liver injury.

**Table 3** and **Figures 2A, B** illustrate the histological findings. Both groups mainly presented with acute hepatitis, with inflammation and necrosis predominantly located in the centrilobular zone III. Mild portal inflammation was observed, consisting chiefly of lymphocytes and neutrophils, along with scattered eosinophils, plasma cells, and KCs. ILICI cases showed more severe lobular inflammation than non-ILICI cases, which was mainly manifested as higher focal necrosis scores (*P* = 0.031) and an increased probability of confluent or bridging necrosis.

**Table 3:** Histological and immunohistochemical characterization.

Histological characteristics	Non-ILICI(n=10)	ILICI(n=19)	*P*-value
Liver injury pattern			0.633
Acute hepatitis	8(80.0%)	17(89.5%)	
Acute cholestasis	1(10.0%)	1(5.3%)	
Chronic hepatitis	1(10.0%)	0(0%)	
Steatohepatitis	0(0%)	1(5.3%)	
Lobular inflammation			
Inflammatory grade^*^			0.321
G2	6(60.0%)	6(31.6%)	
G3	4(40.0%)	11(57.9%)	
G4	0(0%)	2(10.5%)	
Focal necrosis			0.031
1	9(90.0%)	7(36.8%)	
2	1(10.0%)	8(42.1%)	
3	0(0%)	4(21.1%)	
Confluent necrosis			0.464
0	5(50.0%)	7(36.8%)	
1	0(0%)	5(26.3%)	
2	1(10.0%)	1(5.3%)	
3	3(30.0%)	4(21.1%)	
4	1(10.0%)	2(10.5%)	
Bridging necrosis	1(10.0%)	4(21.1%)	0.633
Portal inflammation and interface hepatitis			
Portal inflammation			0.935
0	2(20.0%)	4(21.1%)	
1	6(60.0%)	10(52.6%)	
2	1(10.0%)	4(21.1%)	
3	1(10.0%)	1(5.3%)	
Interface hepatitis			0.673
0	5(50.0%)	11(57.9%)	
1	4(40.0%)	7(36.8%)	
2	1(10.0%)	0(0%)	
3	0(0%)	1(5.3%)	
Ductular reaction	3(30.0%)	7(36.8%)	1.000
Cholestasis	2(20.0%)	1(5.3%)	0.267
Duct injury	7(70.0%)	13(68.4%)	1.000
Fibrosis stage			0.765
0	6(60.0%)	9(47.4%)	
1	2(20.0%)	7(36.8%)	
2	2(20.0%)	2(10.5%)	
3	0(0%)	1(5.3%)	
Sinusoidal fibrosis	1(10.0%)	4(21.1%)	0.633
Steatosis	3(30.0%)	3(15.8%)	0.633
Endothelialitis	0(0%)	1(5.3%)	1.000
CD4^+^T/CD3^+^T(%)	16.9 ± 12.2	18.8 ± 12.7	0.763
CD8^+^T/CD3^+^T(%)	53.0 ± 21.6	59.7 ± 18.4	0.321
PD-1–positive hepatic-infiltrating lymphocytes	6(60.0%)	16(84.2%)	0.193
PD-1 expression level			0.306
Negative	4(40.0%)	3(15.8%)	
Weak positivity	2(20.0%)	9(47.4%)	
Moderate positivity	4(40.0%)	6(31.6%)	
Strong positivity	0(0%)	1(5.3%)	
PD-L1 positivity rate in LSECs, lymphocytes, and Kupffer cells (%)	20.0(8.8, 25.0)	60.0(35.0, 80.0)	0.007
PD-L1 expression level			0.415
Negative	0(0%)	1(5.3%)	
Weak positivity	6(60.0%)	5(26.3%)	
Moderate positivity	3(30.0%)	9(47.4%)	
Strong positivity	1(10.0%)	4(21.1%)	

ILICI, immune-mediated liver injury caused by checkpoint inhibitors; PD-1, programmed cell death-1; PD-L1, programmed death-ligand 1; LSECs, liver sinusoidal endothelial cells.

*: The inflammatory grade was assessed according to the modified Scheuer histological scoring system.

Hepatotoxic agents in the ILICI group included cadonilimab, sintilimab, tislelizumab, camrelizumab, pembrolizumab, toripalimab, ipilimumab, recombinant humanized anti-B- and T-lymphocyte-associated (anti-BTLA) monoclonal antibody, and programmed death-1/interleukin-2α-biased bispecific fusion protein. Those in the non-ILICI group comprised cetuximab, 5-fluorouracil, human epidermal growth factor receptor-2 antibody-drug conjugate (HER-2 ADC), oxaliplatin, capecitabine, sacituzumab govitecan, axitinib, apatinib, CD3-claudin 18.2 bispecific antibody, phosphoinositide 3-kinase delta (PI3Kδ) inhibitor, and cyclin-dependent kinase 4/6 (CDK4/6) inhibitor.

Percentages may not total 100 because of rounding.

**Figure 2:**
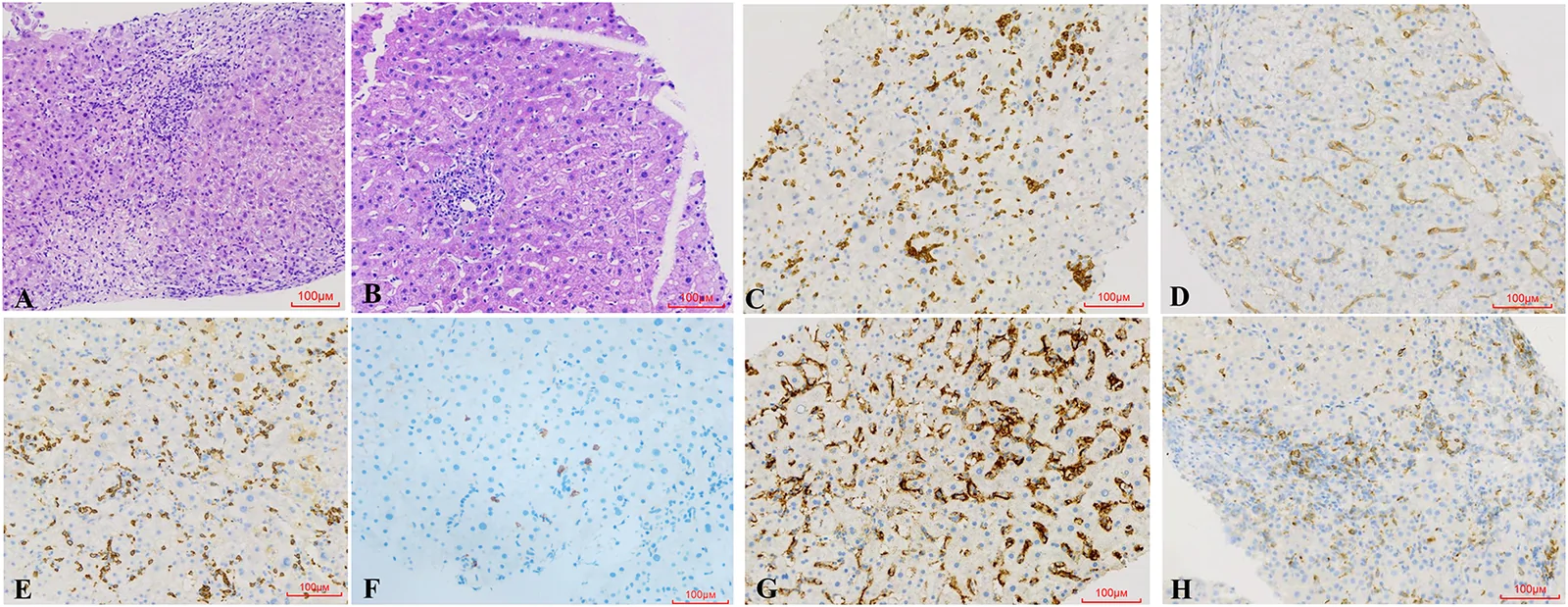
Histological and immunohistochemical characterization of immune-mediated liver injury caused by checkpoint inhibitors (ILICI) and non-ILICI. **(A)** A case of ILICI presents marked lobular necroinflammation accompanied by confluent necrosis (H&E, Scale bar, 100 µm). **(B)** In a case of non_ILICI, lobular necroinflammation is mild and predominantly characterized by spotty or focal necrosis (H&E, Scale bar, 100 µm). **(C)** CD3 immunohistochemical staining of liver tissue from a case of ILICI (Scale bar, 100 µm). **(D)** CD4 immunohistochemical staining of liver tissue from a case of ILICI (Scale bar, 100 µm). **(E)** CD8 immunohistochemical staining of liver tissue from a case of ILICI (Scale bar, 100 µm). **(F)** CD20 immunohistochemical staining of liver tissue from a case of ILICI (Scale bar, 100 µm). **(G)** In a case of ILICI, marked PD-L1 expression is seen in liver sinusoidal endothelial cells, lymphocytes, and Kupffer cells (immunohistochemistry, Scale bar, 100 µm). **(H)** In a case of ILICI, hepatic-infiltrating lymphocytes demonstrate marked PD-1 expression (immunohistochemistry, Scale bar, 100 µm).

Immunohistochemistry (**Table 3**; **Figures 2C–H**) revealed that CD8^+^T cells dominated the infiltrate in both cohorts, with fewer CD4^+^T cells and B cells; CD8^+^T cell counts were numerically higher in ILICI (*P* = 0.321). The inter-observer agreements between the two pathologists for PD-L1 staining intensity and positivity grade were moderate to good, with weighted kappa values of 0.64 (95% CI: 0.389–0.891) and 0.501 (95% CI: 0.295–0.707), respectively. PD-L1 positivity in LSECs, lymphocytes, and KCs was significantly higher in the ILICI group (*P* = 0.007). Moreover, 68.4% of ILICI specimens displayed moderate-to-strong PD-L1 staining intensity, a substantially higher proportion than the 40% seen in the non-ILICI group. ROC analyses were used to assess the diagnostic performance of the positive rates of PD-L1 expression in LSECs, lymphocytes, and KCs for identifying ILICI. The results showed an area under the curve (AUC) of 0.811 (95%CI: 0.641–0.980, *P* = 0.007); at an optimal cut-off value of 30.0%, it yielded a sensitivity of 84.2% and a specificity of 90.0%, respectively (**Figure 3A**). After 0.632 bootstrap correction, the AUC was 0.800, sensitivity 76.0% and specificity 90.0%. The median optimal cutoff across 1000 replicates remained stable at 30%, with an optimism bias of 0.011 (**Supplementary Table 1**). To address the potential confounding effects of pre-biopsy corticosteroid use and inactive HBV carrier status on PD-L1 expression, we performed two sensitivity analyses (**Figures 3B, C**). After excluding patients who received corticosteroids before liver biopsy (n = 18) or those with inactive HBV carrier status (n = 24), the AUCs remained > 0.75 (0.765 and 0.825, respectively). The analysis excluding pre-biopsy corticosteroid users showed a trend toward statistical significance (*P* = 0.058), likely owing to the reduced sample size, but the effect direction was consistent with that of the overall cohort. In addition, Firth penalized logistic regression (**Supplementary Table 2**) showed that, after simultaneously adjusting for pre-biopsy corticosteroid use and inactive HBV carrier status, the PD-L1 positivity rate remained independently associated with ILICI (OR = 1.042, 95% CI: 1.006–1.090, *P* = 0.019). Results were consistent when adjusting for pre-biopsy corticosteroid use alone (OR = 1.043, *P* = 0.018) or inactive HBV carrier status alone (OR = 1.048, *P* = 0.003), with highly stable OR values across all three models (1.042–1.048). Furthermore, we performed a sensitivity analysis restricted to liver-biopsy patients with RUCAM scores ≥6. In this subgroup, the AUC of the ROC curve for intrahepatic PD-L1 expression to discriminate ILICI from non-ILICI was 0.812 (95% CI: 0.615–1.000, *P* = 0.015) (**Figure 3D**). Collectively, these results indicate that PD-L1 expression retains good discriminatory performance for ILICI after adjusting for pre-biopsy corticosteroid use and inactive HBV carrier status, as well as after exclusion of cases with only possible causality as assessed by RUCAM. In parallel, PD-1-positive lymphocytes were more frequent in ILICI patients (84.2% vs 60.0%); this difference exhibited a trend consistent with that of PD-L1 expression, despite the absence of statistical significance (*P* = 0.193).

**Figure 3:**
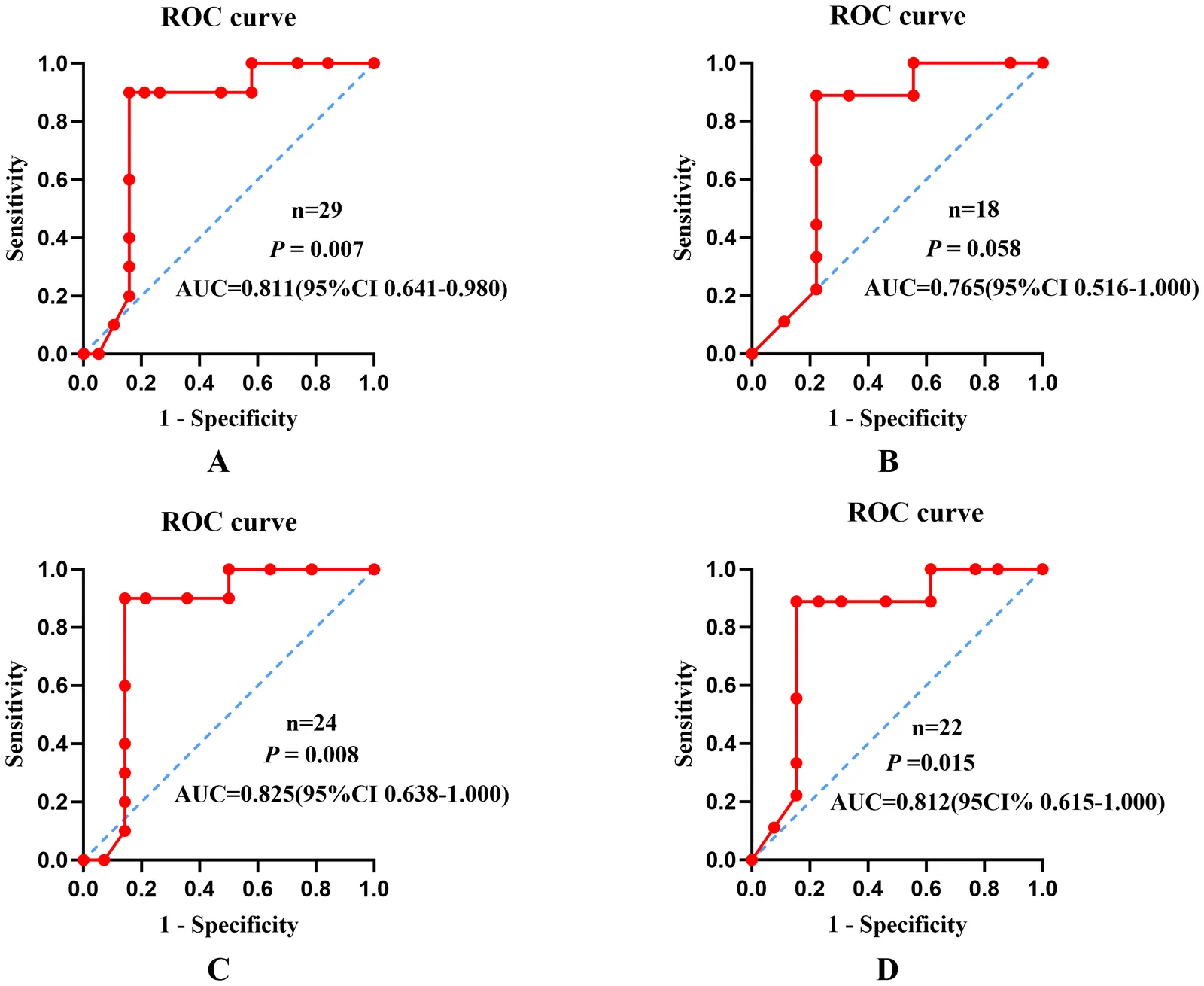
Receiver operating characteristic (ROC) curves evaluating the diagnostic performance of PD-L1 positive expression rates in liver sinusoidal endothelial cells, lymphocytes, and Kupffer cells for immune-mediated liver injury caused by checkpoint inhibitors (ILICI). **(A)** Overall cohort (n = 29); **(B)** Excluding patients receiving corticosteroids before liver biopsy (n = 18); **(C)** Excluding patients with chronic HBV infection (n = 24); **(D)**Excluding cases with only possible causality as assessed by the Roussel Uclaf Causality Assessment Method (n = 22).

To explore the relationship between intrahepatic CD8^+^T cell infiltration and PD-L1 expression, we analyzed the correlation between the CD8^+^T/CD3^+^T ratio and PD-L1 positivity rate in ILICI patients (n = 19). Spearman correlation analysis showed a positive correlation trend between the CD8^+^T/CD3^+^T ratio and PD-L1 positivity rate (r = 0.418, *P* = 0.075). Given that patients with low-grade liver injury had minimal inflammation and comprised only 2 cases, we further performed a sensitivity analysis in patients with high-grade liver injury (n = 17), which showed that this association was strengthened and reached borderline significance (r = 0.471, *P* = 0.056).

We compared baseline characteristics, liver injury severity, treatment regimens, and clinical outcomes between patients receiving liver biopsy (n = 29) and those without biopsy (n = 38) to examine potential selection bias in the liver biopsy cohort (**Supplementary Table 3**). The two groups were comparable with respect to most baseline variables, treatment strategies, and clinical outcomes. However, patients undergoing liver biopsy had a higher proportion of high-grade liver injury and more frequent corticosteroid administration, and baseline hypertension and alcohol history were imbalanced between the groups. These differences are consistent with real-world clinical practice, in which liver biopsy tends to be reserved for more refractory cases. Notably, within the liver-biopsied subgroup, hypertension and alcohol history were well-balanced between ILICI and non-ILICI cases.

### 3.4 Treatment and outcome

Six (24.0%) ILICI patients with high-grade liver injury did not receive corticosteroid treatment: five achieved normalized liver function, while one improved to grade 1 but developed chronic persistent liver enzyme elevation after resuming antitumor therapy. Corticosteroid treatment was administered to 70.0% of ILICI patients and 5.4% of non-ILICI patients (*P* < 0.001), with no statistically significant difference in the initial corticosteroid dosage between the two groups (*P* = 0.454) (**Table 4**). Three ILICI patients received second-line immunosuppressive therapy with mycophenolate mofetil following poor response to corticosteroid treatment.

**Table 4:** Treatment and outcome of liver injury.

Treatment and outcome	ILICI(n=30)	Non-ILICI(n=37)	*P*-value
Treatment			
Corticosteroids	21(70.0%)	2(5.4%)	<0.001
Time from onset of liver injury to initiation of corticosteroid therapy (days)	9.0(1.0, 20.5)	23.0(11.0, NA)	0.250
Average starting dose of corticosteroids(mg/kg)^*^	1.0 ± 0.4	0.8 ± 0.4	0.454
Other therapy			
Mycophenolate mofetil	3(10.0%)	0(0%)	0.169
Immunoglobulin	2(6.7%)	0(0%)	0.197
Plasma exchange therapy	2(6.7%)	0(0%)	0.197
Ursodeoxycholic acid	14(46.7%)	12(32.4%)	0.234
Time to liver enzyme recovery to CTCAE grade 1 (days)^#^	25.0(18.5, 51.0)	20.0(10.0, 53.0)	0.285
Time to the return of liver enzymes to normal or baseline levels(days)^##^	55.5(42.0, 76.5)	57.5(21.5, 124.5)	0.959
Outcome			
Liver enzymes return to normal or baseline levels	26(86.7%)	28(75.7%)	0.391
Death	3(10.0%)	4(10.8%)	
Chronicity	1(3.3%)	5(13.5%)	
Liver failure	3(10.0%)	0(0%)	0.169
Liver enzymes return to CTCAE grade 1^#^	26(89.7%)	23(100%)	0.322

*: The corticosteroid dosage was converted to the equivalent dose of methylprednisolone.

#: Among patients with liver injury of CTCAE grade 2 or higher at onset, including 29 ILICI patients and 23 non-ILICI patients, we assessed the rate of liver enzyme recovery to CTCAE grade 1 and the time to recovery.

##: Calculation of time to the return of liver enzymes to normal or baseline levels was restricted to patients who achieved this recovery endpoint.

ILICI, immune-mediated liver injury caused by checkpoint inhibitors; NA, not available; CTCAE, Common Terminology Criteria for Adverse Events.

All patients in the non-ILICI group had liver function restored to grade 1 or below; four died of tumor progression rather than liver injury. Three patients in the ILICI group developed liver failure and succumbed to concurrent liver failure and tumor progression. In addition, 1 ILICI patient and 5 non-ILICI patients had persistent abnormal liver function for more than 6 months. These patients resumed anti-tumor therapy after partial liver function recovery due to clinical needs, making it difficult to attribute the chronic enzyme elevation to residual liver injury from prior therapy, new hepatotoxicity from retreatment, or a combination of both. The speed of liver function recovery did not differ significantly between the two groups (*P* > 0.05; **Table 4**).

## 4 Discussion

This study was a retrospective cohort analysis that thoroughly compared the clinical and pathological features, treatment approaches, and outcomes of liver injury associated with ICIs versus non-ICIs anticancer drugs. Notably, we discovered a potential diagnostic biomarker, providing a novel approach to identify ILICI.

Our findings indicated that ILICI patients experienced more severe liver injury and a greater requirement for corticosteroids and immunosuppressive agents compared to non-ILICI patients. This was consistent with previous studies, which had suggested that ICIs are more likely to cause high-grade liver injury than chemotherapy (14, 15). The mechanisms behind liver injuries reveal key differences in treatment strategy. Immune-mediated liver injury, like ILICI, arises mainly from T cell activation and cytokine storms (19). In contrast, chemotherapy-related liver injury is caused by direct toxicity, metabolic byproducts, or oxidative stress (12). Consequently, the varying demands for corticosteroids and immunosuppressants highlight the need for personalized treatment approaches. In our cohort, 24.0% of patients with high-grade ILICI recovered to grade 1 or below without corticosteroids. There is growing evidence that some patients with ILICI, including those with grade 3-4, may not require corticosteroids and that hepatitis can resolve spontaneously (20–22). Furthermore, corticosteroids are associated with multiple adverse effects such as increased infection risk, osteoporosis, and immunosuppression. Therefore, it is necessary to optimize treatment strategies and steroid administration criteria for ILICI.

Liver biopsy offers valuable data to identify the drugs responsible for liver injury. Anti-cytotoxic T-lymphocyte-associated protein 4 hepatitis presents with granuloma and fibrin deposition, while anti-PD-1/PD-L1-associated hepatitis is non-granulomatous lobular hepatitis (23). Oxaliplatin may also induce sinusoidal injury and subsequent sinusoidal obstruction syndrome (24). Our study found that, compared with non-ILICI patients, those with ILICI exhibited more severe lobular inflammatory necrosis, higher focal necrosis scores, and more frequent confluent and bridging necrosis. Another trend worth noting was that positive PD-1 expression in hepatic lymphocytes was more prevalent in ILICI patients than in non-ILICI patients (*P* = 0.193), as was positive PD-L1 expression in LSECs, lymphocytes, and KCs (*P* = 0.007). While the magnitude of the difference in PD-1 expression did not meet statistical thresholds, the elevation of PD-L1 expression in ILICI patients showed marked statistical significance. This consistency in trend underscores that the PD-1/PD-L1 pathway may be involved in the hepatic immunopathology of ILICI, as confirmed by Miro Saarela’s team’s research. They found that patients treated with pembrolizumab or nivolumab had higher hepatic PD-L1 levels than those with normal livers, NASH, or PBC; additionally, patients with severe liver-related irAEs showed a significant increase in PD-1-positive cells, while no such increase was observed in patients without liver-related irAEs (25). PD-1 is a type I transmembrane glycoprotein localized to the plasma membrane, which is primarily expressed in activated T cells, B cells, natural killer cells, dendritic cells (DCs), and monocytes (26). PD-1 typically interacts with its ligands to transmit immunosuppressive signals, including PD-L1 and PD-L2. Among them, PD-L1 is primarily expressed on professional antigen-presenting cells (APCs) such as activated T cells, B cells, monocytes, DCs, and macrophages (27); it has also been observed to be upregulated in hepatocytes, KCs, and LSECs under conditions of liver inflammation (28, 29). The binding of PD-1 to PD-L1 inhibits T cell activation, proliferation, and pro-inflammatory cytokines secretion (including interferon-gamma [IFN-γ], tumor necrosis factor-alpha [TNF], and interleukin [IL]-2) (30), thereby inducing immune tolerance in the liver. Before liver transplantation in mice, PD-L1 was expressed only on CD11c^+^ DCs and CD31^+^ sinusoidal endothelial cells, but not on hepatocytes. Its expression increased in all three cell types after transplantation. In livers from PD-L1-knockout donors, liver injury and CD8^+^ T cell infiltration were markedly aggravated, suggesting the PD-1/PD-L1 axis restrains hepatic damage (31). As noted above, ILICI is partly driven by excessive T-cell activation. Persistently activated T cells upregulate PD-1 expression (32), which interacts with upregulated hepatic PD-L1 to protect the liver from autoimmune injury. We also performed quantitative immunohistochemical analysis of PD-L1 positivity rates in LSECs, lymphocytes, and KCs in the ILICI and non-ILICI groups, which showed good discriminatory performance for ILICI (AUC = 0.811, optimal cutoff = 30%). However, it should be noted that pre-biopsy corticosteroid use may suppress hepatic inflammatory infiltration and downregulate PD-L1 expression, potentially underestimating PD-L1 levels in the ILICI group; in addition, inactive HBV carrier status itself may upregulate hepatic PD-L1 expression, and the higher prevalence of HBV carriers in the ILICI group may confound the results. To address these issues, we performed sensitivity analyses by excluding relevant patients and employed Firth penalized logistic regression to adjust for these factors; both showed that the association between PD-L1 and ILICI remained significant with stable effect estimates, suggesting that this association is not entirely driven by the aforementioned confounders. Given the limited sample size, further studies with larger cohorts are needed to confirm these findings.

The liver tissues of ILICI patients were predominantly infiltrated by CD8^+^T cells, with a few CD4^+^T cells and B cells, indicating that CD8^+^T cells are central to ILICI pathogenesis. The pathogenesis of ILICI remains incompletely understood; the current consensus can be summarized into two principal mechanisms. First, ICIs significantly enhance CD8^+^T cells’ activity by blocking inhibitory signals. While CD8^+^T cells attack tumors, tumor and self-antigens are released. The antigens are taken up by APCs, cross-presented, and trigger secondary T cell responses directed at novel epitopes, a process known as epitope spreading that is considered central to irAEs (33). Second, ICIs can reshape T cell subsets: on one hand, they reduce the number of regulatory T cells and downregulate the secretion of anti-inflammatory cytokines such as IL-10 and transforming growth factor-β (34); on the other hand, they regulate the recruitment of T helper (Th) cell subsets, including Th1, Th2, Th17, and T follicular helper cells (Tfh) (35, 36). Dulos et al. observed that anti-PD-1 therapy in prostate cancer and advanced melanoma patients skews immunity toward a Th1/Th17 profile (elevated IFN-γ, IL-2, TNF-α, IL-6, and IL-17) and dampens Th2 activity (reduced IL-5 and IL-13) (35). Recent animal experiments have revealed that anti-PD-1 therapy expanded Tfh cells in a murine non-small-cell lung cancer model (37). IL-21 secreted by Tfh cells not only drives B cell proliferation and antibody affinity maturation but also amplifies the antitumor activity of CD8^+^T lymphocytes (38). Consequently, the Tfh surge triggered by ICIs simultaneously boosts humoral and cellular immune responses, thereby precipitating irAEs (36).

This study has several key strengths. We enrolled 29 patients with liver pathological data, a relatively large cohort in this field. To our knowledge, this is the first study to systematically evaluate the diagnostic value of hepatic PD-L1 expression in ILICI. Our findings suggest that the PD-1/PD-L1 pathway plays distinct roles at different stages of ILICI: initially, PD-1/PD-L1 blockade relieves T cell suppression and mediates liver injury, as evidenced by elevated CD8^+^T cells observed in the ILICI group; subsequently, hepatic PD-L1 upregulation may represent a compensatory feedback to inflammation. As shown in the results section, CD8^+^T/CD3^+^T cells showed a positive correlation trend with PD-L1 positivity, with a strengthened association in high-grade injury, providing preliminary support for compensatory PD-L1 upregulation. The study clarifies the pathological pathogenesis of ILICI and provides a basis for further molecular research.

Our study has some limitations. First, some patients had already received corticosteroids before liver biopsy, which may have affected histologic findings. Ideally, biopsy should precede therapy, yet severe liver injury or concurrent other irAEs often makes this impractical. Second, as a single-center retrospective study, it is subject to some potential biases, including follow-up-driven information bias and liver-biopsy-related selection bias. Frequent weekly laboratory monitoring, driven by short treatment cycles and potential hepatorenal toxicity of anticancer agents, mitigates the former. Given its invasiveness, liver biopsy was largely performed in refractory cases, so the latter cannot be fully eliminated. Compared with non-biopsied patients, biopsied subjects exhibited higher-grade liver injury, more corticosteroid use, and imbalanced baseline hypertension and alcohol history. Although hypertension and alcohol history were balanced between ILICI and non-ILICI cases within the biopsy subgroup, and no robust evidence supports them as confounders for hepatic PD-L1 expression, residual confounding cannot be excluded. Therefore, our PD-L1 findings should be generalized to non-biopsied patients with caution. Third, although the 29-patient liver biopsy cohort represents a relatively large sample in the field of ILICI research, the sample size remains insufficient for robust multivariable analysis. We employed Firth penalized logistic regression, which is suitable for small samples, to reduce separation bias; however, the two binary variables (pre-biopsy corticosteroid use and inactive HBV carrier status) had extremely wide confidence intervals due to few positive events and quasi-complete separation. In addition, even though no significant inter-group differences were detected for global histological inflammatory grade, residual confounding cannot be fully excluded given the limited statistical power and imbalanced focal necrosis between groups. Therefore, the diagnostic value of PD-L1 for ILICI requires further validation in larger external cohorts.

## 5 Conclusions

In summary, compared with non-ILICI patients, those with ILICI exhibited more severe hepatic inflammatory necrosis and more frequently required immunosuppressive therapy. Besides, the PD-L1 positivity rate >30% in LSECs, lymphocytes, and KCs may serve as a potential diagnostic biomarker for ILICI, distinguishing it from liver injury induced by other anticancer agents, but requires further validation in larger external cohorts.

## Data Availability

The raw data supporting the conclusions of this article will be made available by the authors, without undue reservation.
