# Supplementary material for: Clinicopathologic characteristics and diagnostic value of PD-L1 in immune checkpoint inhibitor-related liver injury: a single-center retrospective study
Source: Front Oncol. 2026 Oct 7;16:1928835. doi: 10.3389/fonc.2026.1928835 (PMC13682957; doi:10.3389/fonc.2026.1928835)
Supplement: Supplementary file 1 [file Table1.pdf]

Supplementary Table 1. 0.632 bootstrap internal validation of intrahepatic PD-L1 diagnostic performance for ILICI identification

Parameter	Original apparent performance	0.632 bootstrap optimism-corrected performance
Optimal cut-off value	30%	30%(median across bootstrap replicates)
AUC	0.811	0.800(mean of OOB estimates)
Sensitivity	84.2%	76.0%(mean of OOB estimates)
Specificity	90.0%	90.0%(mean of OOB estimates)

Diagnostic performance of intrahepatic PD-L1 positivity was assessed in the liver biopsy cohort (n = 29). 0.632 bootstrap internal validation with 1000 resamples was applied to correct optimism bias.

ILICI, immune checkpoint inhibitor-induced liver injury; AUC, area under the curve; OOB, out-of-bag.

Supplementary Table 2. Multivariate regression analyses adjusted for pre-biopsy corticosteroid use and/or inactive HBV carrier status

Variable	Model 1: adjusted for both pre- biopsy steroid use and inactive HBV carrier status		Model 2: adjusted only for pre-biopsy steroid use		Model 3: adjusted only for inactive HBV carrier status	
	OR (95%CI)	*P*- value	OR (95%CI)	*P*- value	OR (95%CI)	*P*- value
Pre- biopsy corticosteroid use	7.619 (0.942 – 113.398)	0.057	7.705 (0.950 – 116.292)	0.056	—	—
Inactive HBV carrier status	21.747 (1.360 – 3536.336)	0.027	—	—	13.032 (0.916 – 2018.119)	0.060
PD-L1 positivity rate in LSECs, lymphocytes, and Kupffer cells（%）	1.042 (1.006 – 1.090)	0.019	1.043 (1.007 – 1.091)	0.018	1.048 (1.015 – 1.096)	0.003

Firth penalized logistic regression was used for all models to reduce small-sample separation bias.

HBV,Hepatitis B Virus; PD-L1, programmed death-ligand 1; LSECs, liver sinusoidal endothelial cells; OR,Odds Ratio.

Supplementary Table 3. Comparison between liver-biopsied and non-biopsied patients

Variable	Liver-biopsied patients (n=29)	Non-biopsied patients (n=38)	*P*-value
**Baseline characteristics**			
Age (years), median (IQR)	59.0（53.0，65.5）	57.0（49.0，66.3）	0.621
Gender (male)	20(69.0%)	23(60.5%)	0.475
Drinking history	3(10.3%)	14(36.8%)	0.014
Smoking history	6(20.7%)	13(34.2%)	0.224
Hypertension	3(10.3%)	13(34.2%)	0.023
Diabetes	2(6.9%)	6(15.8%)	0.464
Previous hepatic disease			
HBV	5(17.2%)	8(21.6%)	0.657
NAFL	3(10.3%)	3(7.9%)	1.000
Compensated cirrhosis	1(3.4%)	3(7.9%)	0.810
Liver tumor*	9(31.0%)	20(52.6%)	0.077
Cancer types			0.200
Gastrointestinal cancer	12(41.4%)	18(47.4%)	
Melanoma	8(27.6%)	3(7.9%)	
Primary liver cancer	1(3.4%)	5(13.2%)	
Lung cancer	3(10.3%)	3(7.9%)	
Other cancers	5(17.2%)	9(23.7%)	
Tumor stage (TNM stage)			0.184
Ⅱ	2(6.9%)	0(0%)	
Ⅲ-IV	27(93.1%)	38(100.0%)	
ECOG-PS			0.433
0-1	28(96.6%)	38(100.0%)	

2	1(3.4%)	0(0%)	
Liver function tests			
ALT（U/L）	18.0（14.0，30.5）	26.0（14.5，39.0）	0.389
AST（U/L）	25.0（19.5，34.5）	30.0（22.8，42.3）	0.234
ALP（U/L）	84.0（69.5，132.5）	104.0（82.0，140.3）	0.091
GGT（U/L）	35.0（19.5，45.0）	46.0（24.0，154.5）	0.182
TBIL（μmol/L）	10.6（9.2，12.9）	12.1（9.8，15.3）	0.271
DBIL（μmol/L）	2.4（1.8，2.9）	2.3（1.9，3.5）	0.556
**Severity of hepatotoxicity**			＜0.001
CTCAE grade 1-2	4(13.8%)	22(57.9%)	
CTCAE grade 3-5	25(86.2%)	16(42.1%)	
**Treatment**			
Corticosteroids	14(48.3%)	9(23.7%)	0.036
Mycophenolate mofetil	3(10.3%)	0(0%)	0.152
Immunoglobulin	2(6.9%)	0(0%)	0.184
Plasma exchange therapy	1(3.4%)	1(2.6%)	1.000
Ursodeoxycholic acid	15(51.7%)	11(28.9%)	0.058
**Outcome**			1.000
Liver enzymes return to normal or baseline levels	23(79.3%)	31(81.6%)	
Death	3(10.3%)	4(10.5%)	
Chronicity	3(10.3%)	3(7.9%)	
Liver failure	1(3.4%)	2(5.3%)	1.000
Liver enzymes return to CTCAE grade 1[#]	28(96.6%)	21(91.3%)	0.836

*: Liver tumors include primary liver cancer and liver metastases.

#: Among patients with liver injury of CTCAE grade 2 or higher at onset, including 29 liver-biopsied patients and 23 non-biopsied patients, we assessed the rate of liver enzyme recovery to CTCAE grade 1.

CTCAE, Common Terminology Criteria for Adverse Events; IQR, interquartile range; HBV, hepatitis B virus; NAFL, Non-alcoholic fatty liver; TNM stage, Tumor-Node-Metastasis stage; ECOG-PS, Eastern Cooperative Oncology Group Performance Status; ALT, alanine-aminotransferase; AST, aspartate-aminotransferase; ALP, alkaline phosphatase; GGT, gamma-glutamyl transpeptidase; TBIL, total bilirubin; DBIL, direct bilirubin.

Percentages may not total 100 because of rounding.
